# Unraveling the Complexity of Celiac Disease: A Narrative Review of Its Multisystem Nature

**DOI:** 10.3390/medicina62010120

**Published:** 2026-01-06

**Authors:** Maria Rogalidou, Dimitrios Christodoulou

**Affiliations:** 1 Division of Gastroenterology and Hepatology, First Department of Paediatrics, National and Kapodistrian University of Athens, “Agia Sofia” Children’s Hospital, Thivon & Papadiamantopoulou Street, Goudis, 11527 Athens, Greece; 2 Division of Gastroenterology, Medical School of Ioannina, 45500 Mpizani, Greece

**Keywords:** celiac disease, gluten, *HLA-DQ2*, *HLA-DQ8*, autoimmune disorder, extraintestinal manifestations, gluten-free diet, diagnosis, management

## Abstract

Celiac disease is a chronic immune-mediated disorder triggered by the ingestion of gluten in genetically susceptible individuals, primarily those carrying *HLA-DQ2* or *HLA-DQ8* and, in rare cases, *HLA DQ7* alleles. Although traditionally regarded as a gastrointestinal condition, celiac disease is now recognized as a multisystem disorder with a wide range of clinical presentations. It has been described as a “clinical chameleon” due to its variable manifestations, which may include non-specific symptoms, extraintestinal involvement, or even an asymptomatic course, often identified only through screening of high-risk groups. This narrative review provides a comprehensive overview of celiac disease, highlighting recent insights into its pathogenesis, including genetic predisposition, immune mechanisms, and the role of environmental and microbial factors. It emphasizes the importance of recognizing extraintestinal features, outlines current diagnostic approaches and their limitations, and discusses management strategies centered around the gluten-free diet. Furthermore, it explores emerging therapies aimed at improving patient outcomes and reducing dependence on dietary restriction. By synthesizing the latest developments, this review aims to present a fresh perspective on a condition with significant clinical relevance that is evolving.

## 1. Introduction

Celiac disease (CD) is a common autoimmune enteropathy primarily affecting the small intestine, though it can also involve multiple extraintestinal organs. First described by Aretaeus of Cappadocia around the first to second century A.C. as Koiliaki (from the Greek koilia, “abdomen”), the modern term celiac—historically coeliac—derives from this early description. The diagnosis and management of CD have evolved significantly. In 1888, Dr. Samuel Gee provided a detailed clinical description of children, highlighting dietary modification. For decades, diagnosis relied on clinical response to wheat restriction. The advent of small intestinal biopsy in the mid-20th century enabled identification of villous atrophy, followed by serologic tests—including anti-gliadin, anti-endomysial, and tissue transglutaminase antibodies—which improved non-invasive screening. Recognition of genetic predisposition, particularly *HLA-DQ2*, *HLA-DQ8* and in rare cases *HLA DQ7* alleles, further refined risk assessment. Management remains centered on a strict gluten-free diet, though novel approaches, such as enzyme therapy and immune modulation, are under investigation [1,2].

**Figure 1:**
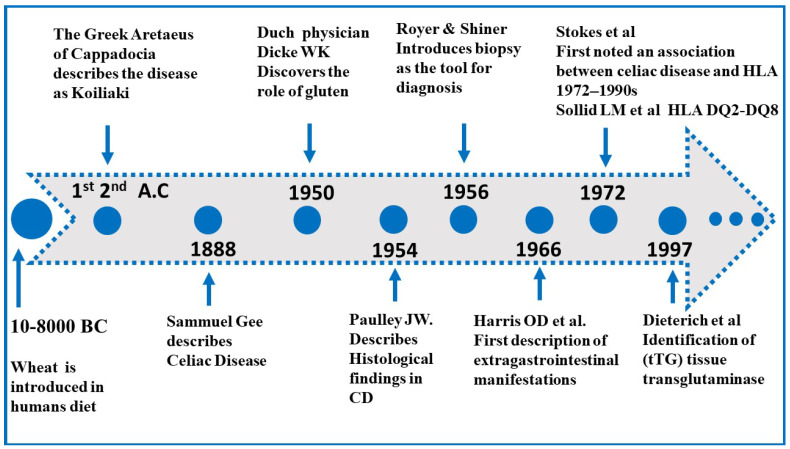
The historical evolution of CD.

The historical evolution [1,2] of CD appears in Figure 1.

CD is unique among autoimmune disorders because its environmental trigger—gluten—is clearly identified. The disease can present as a “clinical chameleon,” with manifestations ranging from classic gastrointestinal symptoms to atypical or extraintestinal features such as anemia, osteoporosis, neurological disorders, dermatitis herpetiformis, recurrent miscarriages, and hepatic abnormalities. Some cases remain asymptomatic and are detected only through high-risk screening [3]. This study provides a comprehensive review of CD, covering its clinical spectrum, diagnostic challenges, epidemiology, advances in monitoring, and emerging therapeutic approaches, aiming to inform more effective detection and management strategies.

## 2. Definition

CD is a chronic, immunologically induced disease that occurs in genetically predisposed individuals (*HLA-DQ2* or *HLA-DQ8*) under the influence of gluten found in cereals (wheat, barley, rye) and is characterized by a wide spectrum of intestinal and extraintestinal manifestations [1,3].

## 3. Pathogenesis

**Figure 2:**
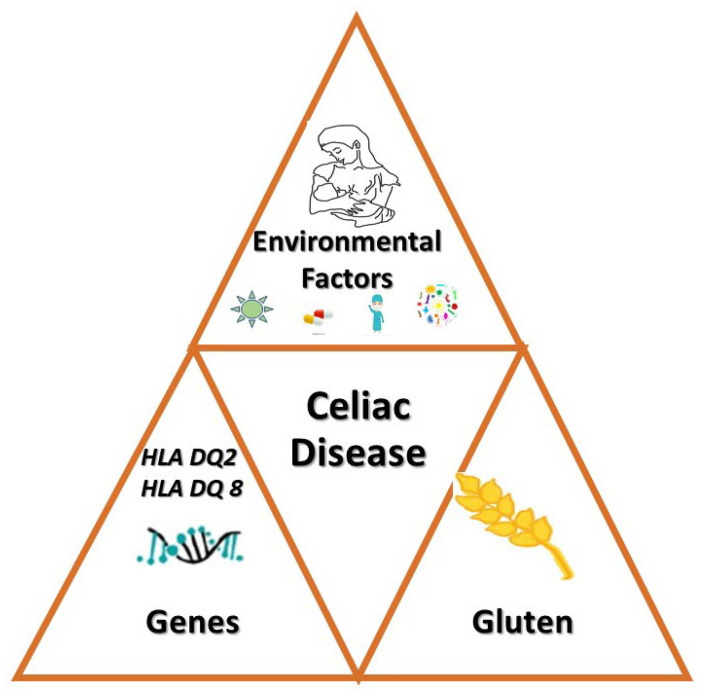
Pathogenesis of CD.

CD results from the interaction between gluten and immune, genetic, and environmental factors. The Pathogenesis of CD appears in Figure 2.

### 3.1. Genetic Factors

CD is a highly heritable chronic inflammatory disorder, with familial occurrence rates of approximately 10–15% and concordance among monozygotic twins reaching 75–80% [4]. Among genetic factors, the human leukocyte antigen (HLA) system represents the primary determinant of disease susceptibility [5,6]. The development of CD is strictly dependent on the presence of *HLA-DQ2* or *HLA-DQ8* alleles, encoded by the *HLA-DQA1* and *HLA-DQB1* genes, which form the α and β chains of the antigen-presenting heterodimer [7,8,9]. Over 90% of affected individuals carry *HLA-DQ2*, with most of the remainder expressing *HLA-DQ8* [6,7]. However, up to 40% of individuals in Europe, the Americas, and Southeast Asia possess these alleles without developing the disease, indicating that *HLA-DQ* expression is necessary but not sufficient and accounts for only about 40% of total genetic risk [5,10].

Homozygosity for *HLA-DQ2.5* confers the highest risk compared with around 3% in heterozygous carriers and is often associated with a more classical and severe clinical phenotype. The most common and high-risk haplotype is *DQ2.5* (DQA1*05:01/DQB1*02:01), while *DQ8.1* (DQA1*03:01/DQB1*03:02) and *DQ2.2* (DQA1*02:01/DQB1*02:02) confer intermediate levels of risk [7,8,9,11]. Rare haplotypes, such as *DQ7.5* (DQA1*05:05/DQB1*03:01), may also contribute when combined with *DQ2.2* in trans, producing a high-risk *DQ2.5*-like genotype [9,11].

Beyond HLA-related factors, genome-wide association studies (GWAS) have identified more than 39 non-HLA loci associated with CD, providing insight into additional immune and molecular pathways involved in disease pathogenesis [4,8,10]. These findings have expanded understanding of the genetic architecture of celiac disease and highlight potential avenues for risk stratification, early diagnosis, and targeted therapeutic approaches [5,8,10].

### 3.2. Gluten

**Figure 3:**
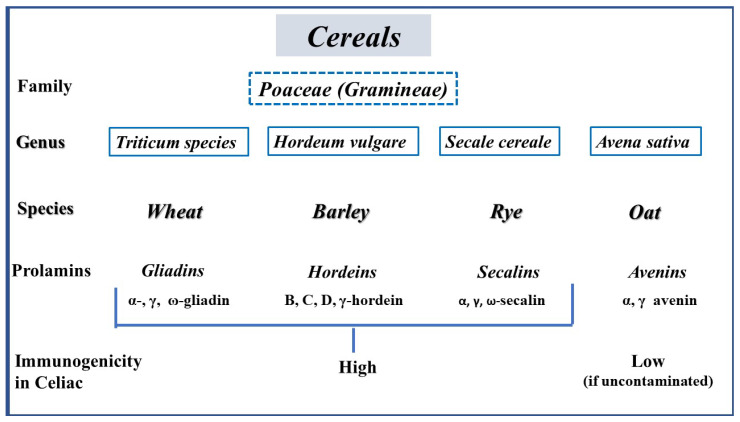
The family tree of cereals, its specific prolamins and their relative immunogenicity.

Gluten (from the Latin “glue”) refers to the prolamin storage proteins found in wheat, rye, barley and oats. The family tree of cereals, showing the specific prolamins they contain and their relative immunogenicity, is illustrated in Figure 3.

Gluten is valued in baking for its elasticity, which makes it particularly useful in bread-making. However, gluten is rich in glutamine and proline residues, which make it resistant to complete digestion by gastric, pancreatic, and brush-border enzymes, resulting in the formation of large peptides among all the remaining peptides with highly variable lengths, the 33mer (αgliadin 57–89) is the most studied one [4,12].

The incomplete digestion of gluten produces a mixture of peptides that can provoke host responses, including increased intestinal permeability and activation of both innate and adaptive immune pathways [13].

A 33–amino acid fragment of A-gliadin (peptide 56–89) is unusually resistant to breakdown by gastrointestinal enzymes and is thought to be a major trigger of the gluten-induced inflammatory reaction in celiac disease [12]. The importance of this enzyme-resistant peptide in disease activity was demonstrated in a phase 2 trial in which 160 patients with celiac disease in remission were challenged with daily gluten; an oral inhibitor targeting its digestion significantly reduced intestinal injury [14]. Similarly, enterocytes from individuals with celiac disease show limited ability to break down another stable fragment, peptide 31–49 of A-gliadin, which does not bind *HLA-DQ2* or *HLA-DQ8*. The resistance of these peptides to proteolysis and their incomplete degradation in celiac patients make them strong candidates as early initiators of both inflammatory and toxic responses. Tissue transglutaminase plays a central role in this process by modifying gliadin peptides in the lamina propria, enhancing their immunogenicity [15].

### 3.3. Environmental Factors

Although the HLA genes required for coeliac disease and gluten consumption are common, the condition develops in only about 1% of the population. This indicates that additional environmental or genetic factors likely contribute to disease onset [16].

#### 3.3.1. The Role of Microbiome

The interplay between genetics, diet, and the gut microbiome plays a central role in CD development and may inform potential preventive or therapeutic strategies. Vaginally delivered, breastfed infants carrying the *HLA-DQ2* genotype—who are at higher risk for CD—exhibit distinct fecal microbiota patterns, suggesting that HLA type influences gut microbial composition [17]. Bacterial diversity also tends to be higher in non-celiac controls than in untreated CD patients, although differences are not always statistically significant [18]. A cross-sectional study indicates that microbiome alterations in CD are not fully restored in all patients after initiating a gluten-free diet (GFD). Healthy adults show fecal microbiota distinct from untreated CD patients, with treatment associated with reduced diversity of *Lactobacillus* and *Bifidobacterium* species, while fecal short-chain fatty acid (SCFA) profiles remain different between patients and controls [19].

Analyses of duodenal and fecal samples from patients with active CD reveal increased *Escherichia coli*, *Prevotella salivae*, and *Neisseria* in the duodenum but not in stool, along with altered microbial protease and peptidase genes, highlighting distinct gut microbial niches and impaired gluten detoxification independent of DQ2 genotype [20]. Further characterization of small intestinal microbiota shows predominance of *Firmicutes*, *Proteobacteria*, and *Bacteroidetes*, with adults harboring 89 genera and children 46, and richness being lower in children. Age and gluten-free treatment appear to shape microbial composition, with principal component analysis indicating both age-related differences and disease effects in adults [21]. Although a definitive microbial signature for CD has not yet been identified, ongoing longitudinal multi-omics studies are expected to provide critical insights for precision medicine and primary prevention strategies [22].

#### 3.3.2. The Role of Infections

Early-life infections may play a role in the development of CD. In a large prospective study of 72,921 children from the Norwegian Mother and Child Cohort Study, children who experienced ≥10 infections in the first 18 months of life had a higher risk of developing CD later in childhood (aOR = 1.32). Specific respiratory and gastrointestinal infections were also associated with increased risk, although surveillance bias and reverse causation cannot be ruled out [23]. Similarly, a study of 1931 genetically at-risk children found that frequent rotavirus infections increased the likelihood of developing CD, even after adjusting for genetics, breastfeeding, and day-care attendance [24,25]. Early rotavirus vaccination, particularly before 3 months of age, reduced the risk of gastrointestinal infections and CD autoimmunity (CDA) in children, especially when gluten was introduced before 6 months (adjusted HR, 0.57). The protective effect was observed in Finland and the United States, but not in countries with low vaccination coverage, such as Sweden and Germany. Gastrointestinal infections increased CDA risk only in unvaccinated children (adjusted HR, 1.46), highlighting the potential role of early vaccination in preventing CDA [25]. In adults, a potential increased risk of CD was observed following Campylobacter infection, but not after *Salmonella* spp., *Shigella* spp., or *Yersinia* spp. infections [26].

Viruses may contribute to CD development, although they are not the sole factor. Specific viral infections can disrupt oral tolerance, particularly in genetically susceptible individuals. Type 1 interferons, including interferon α (IFN-α), play a critical role, as viral infections or gliadin peptides such as P31–43 can trigger inflammatory responses in the gut. Certain viruses, including reoviruses and rotaviruses, may act as triggers and can also influence CD indirectly by altering the gut microbiome. These mechanisms may help explain the variability in symptoms among patients and suggest that multiple pathways contribute to disease onset [27].

In addition, bacterial colonization may influence CD risk. A meta-analysis of 26 studies investigating *Helicobacter pylori* colonization found a significant inverse association with CD, suggesting that *H. pylori* may exert a mild protective effect [28].

#### 3.3.3. The Role of Breastfeeding and Gluten Exposure

Current evidence indicates that breastfeeding does not prevent celiac disease. Large prospective cohort studies, including The Environmental Determinants of Diabetes in the Young (TEDDY) and Celiac Disease Genomic, Environmental, Microbiome and Metabolomic (CDGEMM), as well as randomized trials such as Celiac Disease Primary Prevention with Gluten Introduction Timing (CELIPREV) and Primary Prevention of Celiac Disease in At-Risk Infants (PreventCD), have shown that neither the duration of breastfeeding nor breastfeeding during gluten introduction reduces the risk of developing celiac disease in genetically predisposed children. Recent systematic reviews and updated European Society of Pediatric Gastroenterology, Hepatology and Nutrition (ESPGHAN) guidelines (2023–2024) confirm that early feeding practices, including timing of gluten introduction while breastfeeding, do not modify disease risk. However, breastfeeding continues to provide numerous well-established health benefits, such as immune support and optimal nutrition, independent of any effect on CD. Therefore, while breastfeeding is strongly recommended for general infant health, it should not be considered a preventive measure for CD [29,30,31,32,33,34].

#### 3.3.4. The Role of Timing and Amount of Gluten in Introduction

Current evidence indicates that the timing of gluten introduction in infancy does not significantly alter the long-term risk of developing CD. Guidelines from ESPGHAN [29] recommend introducing gluten between 4 (>17 weeks) and 12 months of age, as studies, including randomized trials and large cohort studies, have shown no protective effect from early or delayed introduction. Introducing gluten before 4 months is discouraged, while introduction after 12 months may modestly increase early celiac autoimmunity, but not overall lifetime risk. Observational data suggest that the amount of gluten consumed in early childhood may influence risk more than the exact timing of introduction. Therefore, gluten can be introduced gradually during complementary feeding, without expecting this to prevent CD, while maintaining normal breastfeeding practices and balanced nutrition [29,30,31,32,33,34,35,36,37].

The amount of gluten an infant consumes in early childhood may influence the development or timing of CD, although research findings are mixed. In a large international study involving over 6600 genetically at-risk children, higher gluten intake during the first five years was linked to increased development of celiac autoimmunity and confirmed CD [38]. Similarly, a Norwegian birth cohort found that consuming more gluten at 18 months of age raised the likelihood of later CD [39]. However, another study reported that gluten intake between 11 and 36 months did not significantly alter disease risk [40]. These observations suggest that early gluten exposure could affect disease onset, but long-term studies are required to understand its impact on lifetime risk.

#### 3.3.5. Early Life Environmental Risk Factors

Maternal nutrition may impact the development of the infant’s immune system and the risk of CD. Analysis of the Norwegian Mother, Father, and Child Cohort Study (MoBa, n = 85,898) showed that higher fiber intake during pregnancy was linked to a lower risk of CD in children, whereas greater maternal gluten consumption was associated with a higher risk. These relationships were largely independent of the child’s gluten intake at 18 months. In a smaller cohort of 149 mother–child pairs, maternal fiber intake did not appear to influence infant gut microbiome diversity or levels of short-chain fatty acids. Overall, these findings indicate that a diet high in fiber and low in gluten during pregnancy may help reduce CD risk, though the precise mechanisms remain uncertain [41].

Changes in gut microbiota and immune markers during early life may precede CD development in genetically susceptible infants. In a nested case–control study, infants who later developed CD exhibited lower bacterial diversity, reduced sIgA levels, and higher abundances of *Bifidobacterium breve* and *Enterococcus* spp., while healthy controls showed increasing Firmicutes diversity, higher TNF-α, and more Bifidobacterium longum. These results indicate that early deviations in microbiota composition and immune maturation may contribute to CD risk, though larger studies are required to validate these findings [42].

#### 3.3.6. Other Factors

Several early-life and environmental factors have been associated with an increased risk of developing CD, although evidence is sometimes conflicting. Studies suggest that season of birth [43,44] and elective cesarean section [45,46] may influence disease risk, though the effect of cesarean delivery is not consistently observed. Additionally, early exposure to antibiotics [47] and proton pump inhibitors [48] has been linked to a higher likelihood of later CD, potentially by altering gut microbiota. Some evidence also points to in utero exposure to maternal iron supplementation as a potential risk factor [49]. These observations highlight that, beyond genetic predisposition, a range of perinatal and early-life environmental exposures may modulate CD risk.

#### 3.3.7. The Role of Mucosa Immune Response

In CD, ingestion of gluten triggers a complex immune response in the small intestinal mucosa. Both the adaptive and innate immune systems are involved. In the adaptive response, gliadin-specific CD4+ T cells in the lamina propria recognize gluten-derived peptides presented by *HLA-DQ2* or *HLA-DQ8* molecules in lamina propria dendritic cells. These T cells, together with macrophages, miofibroblasts and other cells, release pro-inflammatory cytokines and tissue-damaging mediators, such as metalloproteinases, which contribute to crypt hyperplasia and villous atrophy. Concurrently, gliadin also activates the innate immune system within the epithelium. Enterocytes increase expression of interleukin-15 (IL-15), which stimulates intraepithelial lymphocytes (IELs) expressing the activating receptor NKG2D. These cytotoxic IELs kill stressed enterocytes displaying MIC-A, a stress-induced antigen, amplifying epithelial damage. The combined effect of these responses leads to chronic inflammation, epithelial injury, and impaired nutrient [50,51,52].

**Figure 4:**
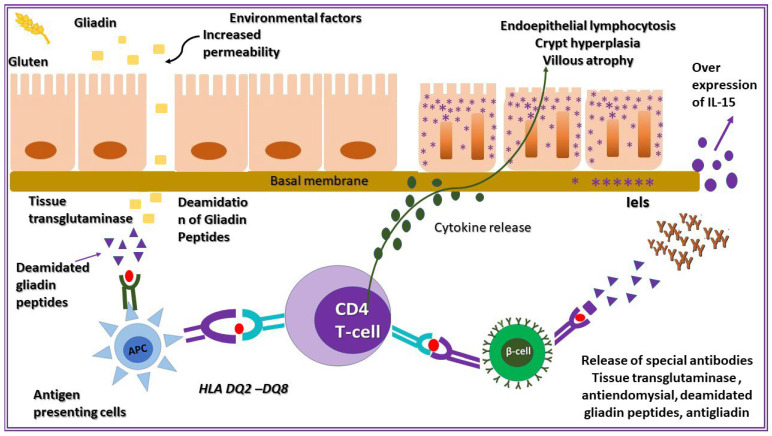
Celiac disease (CD) develops when gluten is broken down in the gut into gliadin peptides that activate the immune system. In the intestinal epithelium, gliadin causes cellular stress and increases the production of interleukin-15, which in turn activates intraepithelial lymphocytes to become cytotoxic and destroy stressed enterocytes. Infections or conditions that increase intestinal permeability allow more gliadin to pass into the lamina propria, where it is deamidated by tissue transglutaminase and presented by *HLA-DQ2* or *HLA-DQ8* molecules to CD4+ T cells. This drives a strong adaptive immune response, with cytokine release and B-cell activation leading to antibody production. Together, the innate and adaptive pathways cause the characteristic mucosal damage of CD, including villous atrophy, crypt hyperplasia, and increased intraepithelial lymphocytosis.

The pathogenesis of celiac disease appears in Figure 4.

## 4. Epidemiology

CD is recognized as a common, long-term condition worldwide. Overall, global patterns of CD tend to reflect the distribution of HLA genotypes associated with susceptibility, provided gluten exposure is present [53]. Differences in access to health care and case-finding methods likely also contribute to the variability observed among populations [54]. Several Western countries have reported a rising incidence of CD, particularly among women and children, a trend likely driven by both environmental changes and improved detection [55]. A pooled global analysis estimates its prevalence at approximately 1.4% in the general population, though this varies by geographic region [56]. In a prospective birth cohort following genetically at-risk infants in Europe and the United States, the incidence reached around 3% in Sweden and 2.4% in Colorado [53,56]. In addition to birthplace, factors such as having a first-degree relative with CD, female sex, and early-life gluten intake influence disease risk. Women are diagnosed at about twice the rate of men, although the exact ratio depends on how cases are identified [57,58]. The condition is also relatively common outside Europe, including North Africa, the Middle East, and South Asia [56,57]. The prevalence of CD varies widely among South American countries. Evidence from a comprehensive systematic review and meta-analysis indicates that the overall prevalence in Latin America ranges from approximately 0.46% to 0.64% [59]. Although previously thought to be rare in Asia, newer evidence suggests otherwise. A meta-analysis found a pooled seroprevalence of about 1.2% in the Asia-Pacific region [60], and a study in China reported a prevalence of roughly 0.76% among adolescents and young adults in wheat-consuming areas [61]. In resource-constrained regions, the actual prevalence may be underestimated because diagnostic testing is limited and other causes of intestinal injury can obscure recognition.

### High-Risk Groups for Celiac Disease

Screening programs using specific serologic markers have shown that the prevalence of CD is significantly higher in certain groups compared with the general population. Due to this elevated risk, routine testing is often recommended for asymptomatic children in these high-risk categories, although the practice remains somewhat debated [62]. Across these high-risk groups, the likelihood of CD is typically 3 to 10 times higher than in the general population.

Individuals in several high-risk categories show a markedly increased prevalence of CD compared with the general population. Screening programs using specific serologic markers have demonstrated elevated rates among first- and second-degree relatives of affected individuals, with estimated prevalence of 7.5% and 2.3%, respectively [63]. CD also occurs more frequently in people with autoimmune or immune-related conditions, including type 1 diabetes, in which up to 16% of children may be affected based on biopsy-confirmed diagnoses [64,65], as well as autoimmune thyroiditis, where 2–7% of patients develop CD [66,67]. Additional associations include juvenile idiopathic arthritis (2–3% prevalence) [68], autoimmune hepatitis (3.6% prevalence) [69], and selective IgA deficiency, in which CD is found in 0.63–9.9% of individuals [70]. Moreover, certain genetic syndromes such as Down syndrome (5–12% prevalence) [71], Turner syndrome (4.5%) [72], and Williams syndrome (up to 10.8%) [73] also carry substantially increased risk. High-risk groups for CD development appears in Table 1.

## 5. Clinical Features

### 5.1. Classic Presentation

**Figure 5:**
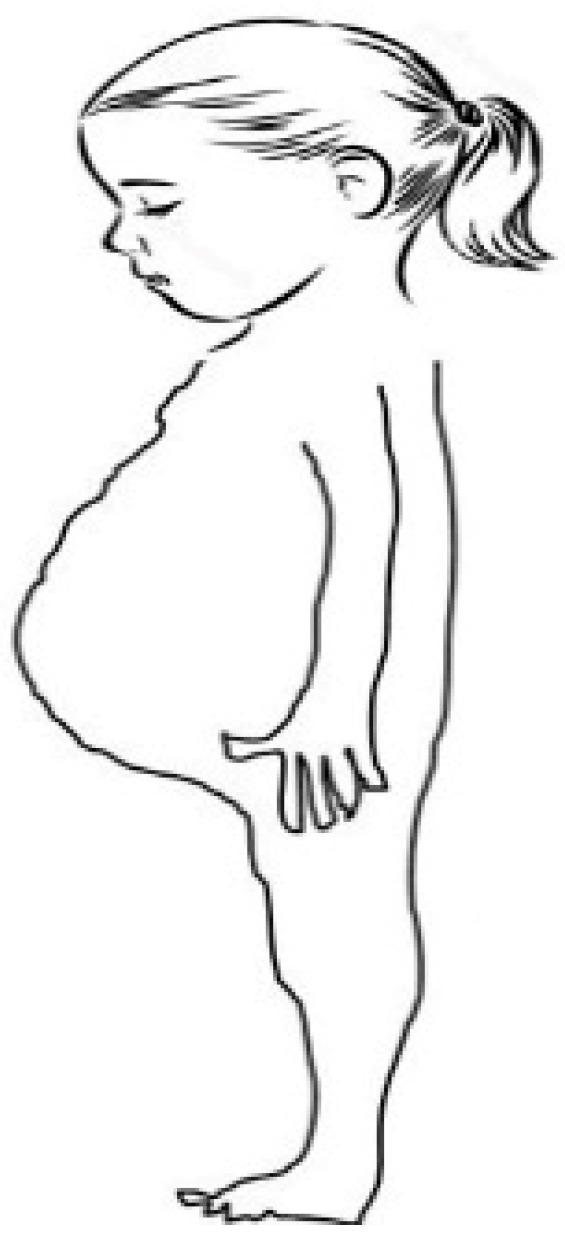
The classical image of celiac disease in children with abdominal distension and malabsorption.

Traditionally, CD was most often identified in children between 6 and 24 months of age, typically after gluten was introduced into their diet [62]. Affected infants commonly developed chronic diarrhea—often producing stools that are bulky, foul-smelling, or prone to floating—as well as poor appetite, abdominal distension, abdominal pain, inadequate weight gain, or weight loss; vomiting may also occur in some cases (Figure 5) [3,17,74]. When the condition went unrecognized, children were at risk of progressing to severe malnutrition. In rare instances, infants with particularly advanced disease could experience a life-threatening state of dehydration and metabolic imbalance, referred to as a celiac crisis. The classic symptoms of CD in children and adults appear in Table 2.

In older children, adolescents and adults, gastrointestinal symptoms tend to resemble those seen in younger patients but are usually less intense. Instead, many individuals in these age groups present with extraintestinal features such as reduced growth velocity, neurologic complaints, or anemia [3,17]. A European collaborative study found that signs of malabsorption were the most frequent gastrointestinal presentation in children younger than three years, whereas abdominal pain was the most common symptom in older children [74]. Interestingly, CD can manifest with either diarrhea or constipation [3,17,75,76], and a significant number of school-age children with the condition are actually overweight or obese instead of malnourished [77].

### 5.2. Atypical/Extraintestinal Manifestations

CD is frequently accompanied by a variety of associated conditions and extraintestinal symptoms, many of which can obscure the clinical picture and delay recognition of the disorder. Some of these manifestations arise directly from autoimmune mechanisms, whereas others result indirectly from chronic inflammation or nutrient malabsorption [78]. According to a retrospective cohort study, 62% of adults newly diagnosed with CD exhibited extraintestinal manifestations, and in 9% of these cases, such features occurred without any gastrointestinal symptoms [79]. When clinicians are unfamiliar with these atypical presentations, diagnosis may be significantly postponed. In one adult cohort, the average time to diagnosis was 2.3 months for patients who had gastrointestinal complaints, compared with 42 months for those who lacked them [80]. Among children, presentation dominated by extraintestinal symptoms has been associated with more severe small-bowel histologic damage at diagnosis [81].

**Figure 6:**
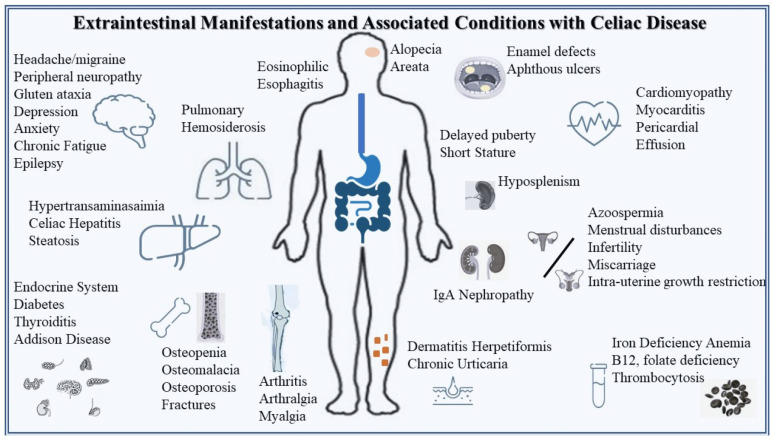
Extraintestinal Manifestations and Associated Conditions with Celiac Disease.

Extraintestinal Manifestations and Associated Conditions associated with CD [78] appear in Figure 6.

A wide range of extraintestinal manifestations of CD has been documented. In some individuals, these non-gastrointestinal features represent the initial or predominant symptoms, and their presence should raise suspicion for CD and lead clinicians to consider appropriate serologic testing.

#### 5.2.1. Extraintestinal Manifestations of CD in Children

**Growth and Development**

Children who develop symptomatic CD frequently experience slowed linear growth. Studies indicate that 8–10% of children labeled as having “idiopathic” short stature actually show serologic markers of CD [82]. In a recent study short stature was found in (20.5%) of children with CD [83]. Notably, this growth impairment can occur even when weight-for-height remains within normal limits and when gastrointestinal symptoms are minimal or absent [84], suggesting that factors beyond simple undernutrition contribute to poor growth. In children with growth delay, catch-up growth is typically rapid during the first year after starting a gluten-free diet and continues for up to three years, although, in some children, catch-up growth may be incomplete [85].

**Delayed Puberty**

In children with untreated CD, delayed puberty is a relatively common extraintestinal feature, often reflecting hypogonadism in girls and androgen resistance in boys [86]. It is estimated that 11–20% of affected pediatric patients experience some degree of pubertal delay [87]. The underlying mechanisms are not fully defined, but are thought to involve a combination of nutritional deficiencies and autoimmune processes directed at hormones, their receptors, or endocrine organs. The prognosis is generally favorable: pubertal progression usually begins within 6–8 months after initiation of a gluten-free diet. If pubertal development does not normalize, an endocrinology evaluation is warranted.

**Behavioral and neuropsychiatric manifestations**

Studies suggest that gluten-related autoimmunity may be linked to subtle behavioral changes in young children. At 3.5 years of age, those with persistently elevated tissue transglutaminase (tTG) antibodies showed more symptoms such as anxiety, low mood, aggression, and sleep problems compared with antibody-negative peers, even though parents were unaware of antibody status at the time of reporting [88]. These differences were not seen by 4.5 years. A school-based study similarly found a modest association between tTG positivity and anxiety [89], while another survey showed that more than one-third of children with CD had clinically significant anxiety or depression, most unrecognized by caregivers [90].

Over long-term follow-up, CD appears to carry a slightly elevated risk of neurobehavioral and psychiatric conditions. Evidence from a large Swedish case–control study shows that individuals diagnosed with CD during childhood had a 1.4-fold higher likelihood of developing disorders such as mood and anxiety conditions, behavioral and eating disorders, attention deficit hyperactivity disorder (ADHD), autism spectrum disorder (ASD), and intellectual disability over a median follow-up of nearly 10 years [91]. Notably, this increased risk was not seen in their siblings, suggesting that shared genetics is unlikely to explain the association.

#### 5.2.2. Extraintestinal Manifestations of CD in Children and Adults

**Oral manifestations**

Dental enamel defects (DED) are significantly more frequent in individuals with CD than in the general population, reflecting disturbances during tooth development likely related to malabsorption or autoimmune mechanisms. A very recent systematic review (2024) found that DED occurs in 50–94% of CD patients, often presenting as symmetrical, chronological defects according to Aine’s classification [92]. Moreover, a comparative study in children and adolescents reported DED in 64.4% of CD patients versus 24.5% of healthy controls [93]. Recognition of these characteristic enamel changes—especially when symmetrical and widespread—can serve as an important clue for early diagnosis of CD, even in the absence of gastrointestinal symptoms [92].

Recurrent aphthous stomatitis (RAS) and painful ulcers are among the most common soft-tissue oral manifestations in patients with CD and appear significantly more often than in the general population. A recent large cohort study of adult CD patients reported that more than half (56%) had symptoms before diagnosis, and that a strict GFD relieved ulcers in about 69% of them [94]. A 2023 systematic review found RAS in about 34.6% of CD subjects included in the analyzed studies [95]. The underlying mechanisms remain unclear but might involve immune-mediated reactions, mucosal-immunity disturbances, and possibly nutritional deficiencies due to malabsorption [96].

**Neurological manifestations**

Neurological symptoms occur in an estimated 6–10% of individuals with CD [76], and rates may reach 42% in untreated cases [97]. Proposed mechanisms include cross-reactive antibodies, immune-complex deposition, direct neurotoxicity, and deficiencies in vitamins or other nutrients [97].

Headaches—particularly migraines—can be an early manifestation of CD and often present with greater intensity, leading to more frequent medical visits [78,98]. The pooled prevalence of headache is about 26% in adults and 18.3% in children with CD [78,98]. Idiopathic, migraine, and tension headaches are more common in CD, whereas no link is seen with cluster headache, hemicrania continua, or trigeminal neuralgia [98]. Encouragingly, both their frequency and severity often improve on a GFD, particularly in children [78,79,98].

Peripheral neuropathy—Individuals with CD have a 2.5–3.4-fold higher risk of peripheral neuropathy than the general population [78,99]. An even more frequent condition, “gluten-induced neuropathy,” involves idiopathic peripheral neuropathy with positive celiac serology but no enteropathy [78]. In CD, neuropathy may precede the diagnosis, appear afterward, or be the sole clinical feature [99]. Symptoms vary by type and may involve painful paresthesia (including facial or oral), mild gait disturbance, and mild limb or focal weakness [78]. Response to a GFD is inconsistent and may depend on adherence, disease duration, and underlying cause; the presence of anti-neuronal antibodies predicts poorer improvement [78].

Gluten ataxia (GA) is a well-described neurological manifestation linked to gluten exposure, with or without enteropathy [100]. It usually appears in mid-adulthood and presents with gait and lower-limb ataxia, dysarthria, nystagmus, and other oculomotor signs [100]. Fewer than 10% have GI symptoms, but about 40% show duodenal changes compatible with CD [78,100]. Anti-gliadin antibodies may be present, whereas tTG antibodies are typically absent in gluten ataxia [78]. The disorder is thought to arise from antibody cross-reactivity with cerebellar Purkinje cells, and tTG6 antibodies—seen in 73% of cases—decline with a GFD [98]. A GFD generally improves symptoms, though recovery may depend on symptom duration due to possible irreversible Purkinje cell loss [100]. In a recent CD cohort, gait instability (24%), gait ataxia (29%), nystagmus (11%), tTG6 positivity (40%), and cerebellar Magnetic Resonance Imaging (MRI) abnormalities were common, with improvement after one year of GFD [97].

Gluten-induced cognitive impairment “Brain fog” describes patient-reported concentration problems, short-term memory issues, word-finding difficulty, and confusion after gluten exposure [101]. Though also seen in conditions like multiple sclerosis and fibromyalgia [101]. Proposed mechanisms include cytokine-driven neuroinflammation, altered blood–brain barrier permeability, reduced tryptophan and serotonin, gluten-derived exorphins, and microbiome changes [101].

Epilepsy—Individuals with CD have an increased risk of epilepsy, most commonly complex partial seizures [78]. A recognized triad includes CD, bilateral parieto-occipital calcifications, and seizures, though seizures do not always originate from the calcification sites, which may instead be linked to headaches [102]. Proposed mechanisms include folic acid deficiency or autoimmune processes, often with white matter abnormalities [102].

**Dermatological manifestations**

While dermatitis herpetiformis (DH) is well known in CD, the link with other skin conditions has been less clear. A Swedish population-based study (1969–2016) including 43,300 CD patients and 198,532 matched controls found a higher long-term risk of skin disorders in CD (HR 1.55). Specific conditions with increased risk included eczema (HR 1.67), psoriasis (HR 1.55), urticaria (HR 1.52), vitiligo (HR 1.90), acne (HR 1.39), and alopecia areata (HR 1.78). This elevated risk persisted over a median follow-up of 11.4 years [103]. Nonspecific rashes have also been linked to gluten exposure [104].

DH—Both diseases occur in gluten-sensitive individuals, share the same HLA haplotypes and improve following a GFD. DH is the most common dermatologic manifestation of CD, presenting as intensely pruritic vesicles and papules on the elbows, knees, and buttocks. Its incidence appears to be declining, likely due to earlier CD diagnosis, with recent Finnish data reporting a prevalence of 4% among untreated CD compared to 17–20% in older cohorts [105]. DH primarily affects adults, typically presenting in the third to fifth decades of life, but it can also occur in children, though less commonly. Pathognomonic granular IgA deposits of anti-transglutaminase 3 in the papillary dermis confirm diagnosis by skin biopsy [105]. Lesions typically respond to a GFD [78,105] though remission may take months or years.

Psoriasis—Psoriasis is more common in CD, with a 1.4–3.1-fold increased risk [78,106]. Some studies suggest a GFD can improve psoriasis, possibly through enhanced vitamin D absorption or improved intestinal barrier function [106]. In some cases, psoriasis may be the sole manifestation of CD [106].

Chronic urticaria—Chronic urticaria (lasting >6 weeks) is more common in CD and associated with HLA-DQ8 (HR 1.92, 95% CI 1.48–2.48) [105]. A GFD can lead to complete remission in some cases, suggesting urticaria may represent a CD manifestation [78,103].

**Ocular Manifestations**

CD can affect the eyes through nutrient malabsorption and immune-mediated mechanisms. Reported manifestations include night blindness, dry eye, cataracts, thyroid-associated orbitopathy, uveitis, central retinal vein occlusion, and neuro-ophthalmic disorders. In some cases, ocular symptoms may be the initial presentation of CD [107].

**Musculoskeletal manifestations**

CD often causes metabolic bone disease, increasing fracture risk. Mechanisms include nutrient malabsorption, inflammation, hypogonadism, and low body weight. Bone density and microarchitecture are reduced, and fracture risk may persist despite a gluten-free diet. Optimal DXA timing and use of osteoporosis medications in CD remain unclear, underscoring the need for standardized management guidelines [108].

Both diagnosed and undiagnosed CD are associated with reduced bone mineral density and increased fracture risk. A nationwide Danish registry study (2000–2018) including 9397 CD patients and 93,964 matched controls found a significantly higher risk of osteoporosis (HR 5.39; 95% CI 4.89–5.95), major osteoporotic fractures (HR 1.37; 95% CI 1.25–1.51), and any fracture (HR 1.27; 95% CI 1.18–1.36). Elevated odds were also observed prior to CD diagnosis. These findings emphasize the need to consider bone health in CD management and highlight the potential benefits of early diagnosis and treatment [109].

Management of bone disease in CD remains inconsistent due to limited long-term data. In a 10-year follow-up study of 107 CD patients with low bone density at diagnosis, T-scores remained mostly stable, with minor changes in FRAX^®^ fracture risk. Six major fractures occurred, and FRAX^®^ predicted risk accurately. The study suggests longer dual-energy X-ray absorptiometry (DEXA) intervals for patients with osteopenia and no risk factors, while maintaining 2-year checks for those with osteoporosis or additional risks [110].

Myalgia is a common early symptom of CD, often linked to nutritional deficiencies or systemic inflammation. In adults, only about half experience improvement after 24 months on a GFD. Other conditions, such as idiopathic inflammatory myopathies and fibromyalgia, can coexist with CD and may not respond to the GFD [111].

Arthralgia and arthritis—Joint pain and arthritis are among the musculoskeletal manifestations that may accompany CD. A recent meta-analysis estimated that about 10.7% (95% CI 6.9–15.1%) of CD patients without other rheumatic disease report joint complaints (arthralgia or arthritis) [112]. Nevertheless, evidence for a statistically increased risk of established arthritis in CD remains weak or inconsistent [113].

**Cardiovascular Manifestations**

Celiac Cardiomyopathy (CCM) is a rare but serious complication of CD that can affect both adults and children. A recent review identified 28 cases, with most adult patients being male and pediatric patients female [114]. Cardiomyopathy often preceded or coincided with CD diagnosis, and diagnosis was delayed by ~16 months on average. Treatment with a gluten-free diet plus standard cardiac therapy led to improvement in 61% of patients, highlighting the importance of early recognition [114].

Early Myocardial Dysfunction in Pediatric Celiac Disease—A meta-analysis of 916 children with celiac disease versus 569 controls found early subclinical myocardial dysfunction, with reduced fractional shortening and myocardial performance index. Routine cardiac monitoring is recommended, though the long-term effects of a gluten-free diet remain unclear [115].

**Pulmonary Manifestations**

Lung involvement in CD is rare. Although an increased risk of chronic obstructive lung disease has been reported, no causal relationship has been established [78]. The only well-documented pulmonary complication is pulmonary hemosiderosis (PH). Lane-Hamilton syndrome, the coexistence of CD and PH, occurs in both children and adults and may present without gastrointestinal symptoms. Immune complex deposition increases alveolar capillary permeability. Diagnosis is usually confirmed via bronchoscopy and small bowel biopsy. Initiating a strict gluten-free diet often leads to clinical improvement, highlighting the importance of CD screening in pediatric PH [78,116]. CD has been linked to a higher risk of certain allergic conditions. In a 2023 case–control study in Zahedan, Iran, involving 173 children with CD and 173 matched healthy controls, asthma (12.1% vs. 5.8%) and allergic rhinitis (29.5% vs. 14.5%) were more common among children with CD, whereas rates of atopic dermatitis were similar between groups (12.1% vs. 9.2%) [117].

**Renal Manifestations**

CD is linked to an increased risk of kidney disorders, including glomerulonephritis and end-stage renal disease. A meta-analysis of nine studies (over 2 million patients) confirmed these associations, highlighting the need for renal monitoring and further research on underlying mechanisms [118]. IgA nephropathy (IgAN), or Berger’s disease, is an immune-complex–mediated glomerulonephritis marked by glomerular IgA deposits, leading to macroscopic hematuria and impaired kidney function. CD may be associated with IgA nephropathy (IgAN) through IgA-tTg deposits. In a case series of nine IgAN patients (four with CD), kidney biopsies showed IgA-tTG deposits in all three CD patients on a gluten-containing diet, but not in a patient on a gluten-free diet. Interestingly, some non-CD IgAN patients also had these deposits, suggesting that while IgA-tTG can occur in IgAN, it is not specific to CD [119].

**Hematologic manifestations**

Anemia is the most common extra-intestinal manifestation of CD, with iron deficiency being the leading cause. Vitamin B12 and folate deficiencies, as well as systemic inflammation, contribute to over 15% of cases [120]. CD is found in 3–5% of patients with iron deficiency anemia [121]. In subclinical CD, iron deficiency anemia is the most frequent manifestation in both adults and children [78] and may persist despite a long-term gluten-free diet. In a study of 311 patients, 17.8% of adults and 4.4% of children still had anemia after 8–10 years of GFD. Delayed diagnosis and severe intestinal damage likely contribute to persistent anemia [122].

Hyposplenism—CD can be associated with hyposplenism [78], though its prevalence varies widely and is rare in children. Clinical signs include thrombocytosis and Howell-Jolly bodies, while imaging and pitted red cell counts can aid diagnosis. Hyposplenism increases susceptibility to infections, particularly pneumococcal disease, and vaccination against pneumococcus [121,123], Haemophilus influenzae, and meningococcus is recommended [78]. The reversibility of hyposplenism with a gluten-free diet remains uncertain.

**Reproductive System Manifestations**

CD can affect reproductive health [78,124]. Evidence on its impact on infertility is mixed: some studies report a higher prevalence of unexplained infertility in CD patients, while others find no significant difference compared to the general population. Untreated CD may contribute to fertility issues through nutritional deficiencies, immune-mediated effects, or sexual dysfunction. Adherence to a GFD may improve fertility outcomes, but further high-quality studies are needed to clarify the relationship and guide screening and management [124].

A recent meta-analysis of 18 studies evaluated pregnancy risks in women with CD and showed that undiagnosed CD increases the risk of miscarriage, fetal growth restriction, stillbirth, preterm delivery, cesarean section, and low birthweight. Early diagnosis and a gluten-free diet reduce these risks, emphasizing timely detection and management in pregnancy [125].

**Psychiatric Manifestations**

CD is associated with an increased risk of anxiety and depression in both children and adults. A meta-analysis found that CD patients have higher odds of anxiety (OR 2.26) and depression (OR 3.36), and adherence to a GFD may improve these symptoms. These findings suggest that psychiatric screening and dietary management should be considered in the care of CD patients, though further research is needed to clarify the underlying mechanisms [126]. Another meta-analysis of 37 studies examining several psychiatric disorders found increased odds of anxiety, depression, eating disorders, ASD, and ADHD in CD patients compared to controls, while no significant association was observed with bipolar disorder or schizophrenia. These findings highlight the need for further research on the biological mechanisms and the potential impact of a gluten-free diet on psychiatric outcomes in CD [127].

**Fatigue**

Fatigue is a common and significant complaint in CD, with prevalence ranging from 8% to 100% and higher severity than in healthy controls. Studies suggest a gluten-free diet may reduce fatigue, though evidence is limited and results are sometimes conflicting [128]. In a study comparing 90 patients with newly diagnosed CD to 90 healthy controls, 41–50% of patients experienced clinically relevant fatigue. Patients had significantly higher fatigue scores on the fVAS, FSS, and inverted SF-36 vitality subscale compared to controls. Fatigue severity was linked to female sex, younger age, and higher pain and depression scores, but not to biochemical variables such as hemoglobin. This highlights that fatigue is frequent and severe in untreated CD [129].

**Digestive System manifestations**

**Hepatic Manifestations**

Autoimmune liver diseases are increasingly recognized in association with CD. Ongoing intestinal inflammation and immune responses directed against gluten and tissue transglutaminase may contribute directly to hepatic injury, leading to abnormalities ranging from isolated transaminase elevations to autoimmune hepatitis [69,78]. A recent systematic review and meta-analysis that included 42 studies and nearly 9000 patients found that liver enzyme elevation occurs in about 21% of individuals with celiac disease, with similar rates in adults and children [130].

Celiac hepatitis accounted for almost half of the identified causes of hypertransaminasemia. Importantly, most patients showed excellent adherence to a gluten-free diet, and over 86% experienced normalization of liver tests after dietary treatment. These findings reinforce that liver dysfunction is common in CD but typically resolves with strict gluten withdrawal [130].

A large population-based study of more than 70 million individuals found that people with CD have a significantly higher prevalence of autoimmune and metabolic liver disorders. Autoimmune hepatitis and primary biliary cholangitis were markedly more common, with adjusted odds over sevenfold and fourfold higher, respectively, and risks were even greater in those positive for anti-tTG antibodies. Primary sclerosing cholangitis and non-alcoholic fatty liver disease (NAFLD) were also more frequent, with NAFLD risk remaining elevated regardless of diabetes type. Overall, the findings show that celiac disease strongly increases susceptibility to several liver diseases, emphasizing the need for routine liver assessment in this population [131].

A systematic review and meta-analysis examined the prevalence of CD in individuals with autoimmune hepatitis (AIH). Analyzing data from eight studies including 567 AIH patients, biopsy-confirmed CD was found in 4.1% of cases, resulting in a pooled prevalence of 3.5%, which is notably higher than the roughly 1% seen in the general population. When studies relying on serology without biopsy were included (total n = 1817), the pooled prevalence was 2.9%. These findings suggest that CD occurs more frequently in AIH patients than in the general population, supporting consideration of CD screening in this group [132].

**Irritable bowel Syndrome**

A Swedish registry-based study found that patients with CD have a substantially increased risk of irritable bowel syndrome (IBS) both before and after CD diagnosis. Among 27,262 CD patients, 2.7% were diagnosed with IBS during an average follow-up of 11 years, compared to 0.9% in matched controls, corresponding to an adjusted hazard ratio (aHR) of 3.11. Risk remained elevated even after 10 years (aHR 2.00) and was higher than in siblings (aHR 2.42). Interestingly, CD patients with persistent villus atrophy were less likely to be diagnosed with IBS than those with mucosal healing. These findings suggest a long-term bidirectional association between CD and IBS, highlighting the need for clinicians to monitor gastrointestinal symptoms in CD patients [133].

**Eosinophilic Esophagitis**

A retrospective cohort study using a large population database found that patients with eosinophilic esophagitis (EoE) have a higher prevalence and incidence of CD, and vice versa. Among 46,398 EoE and 84,383 CD patients, those with both conditions showed increased risks of asthma, allergic rhinitis, atopic dermatitis, rheumatoid arthritis, and iron deficiency anemia. The study highlights that concurrent EoE and CD not only coexist more frequently than expected but also increase the risk of complications, emphasizing the need for careful monitoring in affected patients [134].

The wide spectrum of intestinal and extraintestinal manifestations of celiac disease—from asymptomatic forms to severe clinical disease—reflects variability in genetic background, immune responses, environmental exposures, and host-related factors. Differences in HLA genotype (*DQ2.5* vs. *DQ2.2* or *DQ8*) and non-HLA susceptibility genes influence the strength and quality of gluten-specific immune activation. Heterogeneity in immune regulation, including the magnitude of adaptive T-cell responses, levels of pro-inflammatory cytokines (e.g., IFN-γ, IL-15), and efficiency of regulatory T-cell function, affects the degree of mucosal damage and systemic immune involvement. Environmental modifiers, such as gluten dose and duration of exposure, infections, microbiome composition, intestinal permeability, and age at diagnosis, further shape disease expression. Additionally, nutritional deficiencies, hormonal factors, comorbid autoimmune diseases, and individual tissue sensitivity contribute to extraintestinal manifestations involving the skin, liver, bone, nervous system, and hematologic system. Together, these interacting factors determine whether celiac disease remains clinically silent or progresses to overt, multisystem disease [1,3,16].

## 6. Diagnosis

CD is diagnosed through a combination of duodenal biopsy and clinical response. Biopsy shows intraepithelial lymphocytosis, crypt hyperplasia, and villous atrophy, while seronegativity or a reduction in specific antibody levels and improvement on a strict gluten-free diet confirms the diagnosis and distinguishes it from other causes of intestinal damage [3]. The diagnostic criteria initially developed by the ESPGHAN, over time, have been revised [135]. The Diagnosis usually involves serologic testing for CD-specific antibodies and confirmation by small intestinal biopsy, though a non-biopsy approach may be used in select children under specific rules [76,135,136]. The most recent adult guidelines for celiac disease introduce significant updates, including a conditional no-biopsy diagnostic approach for carefully selected adults who have markedly elevated IgA anti-TG2 levels (≥10× the upper limit of normal) [137].

### 6.1. Serology

Serologic testing has markedly improved the accuracy of CD diagnosis. Modern assays—particularly IgA anti-TG2 and IgA anti-endomysium (anti-EMA)—outperform older tests such as IgA and IgG anti-gliadin antibodies (AGA), which show wide variability and substantially lower diagnostic accuracy and are therefore no longer recommended for routine evaluation [3,17,137,138]. IgA anti-TG2 demonstrates high diagnostic performance (PPV 90%, NPV 98%) with sensitivity and specificity consistently strong across studies, including up to 99% and 100% in automated assays [137,138]. IgA-EMA provides near-absolute specificity (99.6%) but slightly lower sensitivity (88%) [136]. IgA/IgG-deamidated gliadin peptides (DGP) tests show good accuracy and are helpful when anti-TG2 is negative, though isolated DGP positivity has a low predictive value in low-risk patients [137,138].

Serological assays for CD, such as anti-TG2 ELISA tests, can show significant variability in results due to differences in assay design and quality control. The ProCeDE study [139] found up to 20% discordance between local and central lab results. To improve accuracy, assays should be rigorously validated, and a certification system could standardize testing across labs. Point-of-care tests (POCT) are useful for screening but tend to have lower accuracy and are affected by operator technique. Positive POCT results should always be confirmed with formal tests or biopsy [137,140].

According to the latest guidelines for CD [135,137], for the initial testing of CD at any age, it is recommended to use the IgA anti-TG2 antibody as a single test. Total IgA levels should be measured concurrently to rule out IgA deficiency [70,137], and testing should be performed while the patient is on a gluten-containing diet. Additionally, a routine combination of serological tests for the initial diagnosis is not recommended, as it offers minimal additional value and increases both cost and complexity.

### 6.2. Genetic Testing

*HLA-DQ2/8* typing is not routinely used for the diagnosis of CD, but it is recommended in certain situations, such as when there is uncertainty about the diagnosis (e.g., ambiguous results, gluten-free diet started before testing, potential CD, or seronegative cases). May also be recommended for screening high-risk groups for developing CD [135,137].

The combined predictive value of *HLA-DQ2* and *HLA-DQ8* alleles in assessing celiac disease (CD) risk is defined by a high negative predictive value and a low, population-dependent positive predictive value. Over 95% of individuals with CD carry *HLA-DQ2* and/or *HLA-DQ8*, so the absence of both alleles reliably excludes CD in most cases across diverse populations (e.g., >98% of CD patients have these haplotypes [141]. However, these alleles are common in the general population (30–55% in some groups), meaning many carriers never develop disease, which limits positive predictive value [141]. The allelic subtype and gene dose further modify risk, with homozygous or combined high-risk genotypes conferring a stronger association with disease compared to single low-risk variants [142]. Consequently, *HLA-DQ2/DQ8* typing is most useful for excluding CD and stratifying genetic risk in first-degree relatives or diagnostically ambiguous cases, rather than for confirming disease on its own [142].

The genetic risk for celiac disease varies across populations carrying *HLA-DQ2* and *HLA-DQ8*. In Europe, most CD patients carry *DQ2* (~90–93%), with a minority carrying *DQ8* (~5–10%), and these alleles are common in the general population, limiting positive predictive value. In the Americas, *DQ8* is more frequent than in European cohorts due to admixture, producing a more heterogeneous haplotype distribution. In Southeast Asia, *DQ2* frequencies are very low (<5%), and *DQ8* is moderate (~8–10%), contributing to lower genetic susceptibility and overall CD prevalence. These differences highlight how population-specific HLA distributions influence risk assessment and the utility of genetic testing for celiac disease [143,144,145].

### 6.3. The Role of Serologic Testing Versus Genetic Markers

Serologic testing and genetic markers serve complementary roles in identifying individuals at risk for celiac disease. Serologic tests—particularly tTG-IgA and EMA-IgA—have high sensitivity (90–98%) and specificity (95–98%), making them the frontline tools for detecting active disease and guiding the need for biopsy. Their accuracy depends on ongoing gluten intake, and they are also useful for monitoring dietary adherence. In contrast, genetic testing for *HLA-DQ2* and *DQ8* has very high sensitivity (~95–99%) but low specificity (~30–40%), providing a strong negative predictive value but limited positive predictive value. HLA typing is most useful for excluding disease in ambiguous cases, assessing risk in first-degree relatives, or when patients have already initiated a gluten-free diet. Together, serology identifies active disease, while genetic testing stratifies risk and rules out celiac disease, making the combination clinically valuable in selected scenarios. Serology is the frontline tool for detecting active celiac disease and guiding biopsy, with tTG-IgA and EMA-IgA offering high sensitivity and specificity. Genetic testing is primarily a rule-out tool, particularly useful when serology is inconclusive or the patient is already on a gluten-free diet before testing. The optimal strategy often combines the two approaches: serology is used for diagnosis, while HLA typing provides clarification and risk assessment, leveraging its high negative predictive value to exclude disease in ambiguous cases [1,3,16].

### 6.4. Endoscopy

A small-intestine biopsy is still the most reliable way to diagnose CD, and it should be performed whenever the condition is strongly suspected, even if blood tests are negative. This step is important because the diagnosis leads to permanent lifestyle changes, including a restrictive and often costly diet.

For diagnosing CD, at least four biopsies should be taken from the distal duodenum and two from the duodenal bulb, even if the duodenum appears normal endoscopically [137]. This is because mucosal damage in CD can be patchy, and including the duodenal bulb helps detect early or localized involvement, improving diagnostic yield by about 6.9% [140]. Using single-bite biopsy forceps is recommended for optimal sample orientation [137].

Endoscopic signs suggestive of CD include a thin, flattened mucosa, reduced duodenal folds, fissures, nodules, scalloping, a mosaic pattern, and increased visibility of submucosal vessels. While these features are well-documented, it is important to note that the appearance can sometimes be completely normal, making diagnosis challenging based solely on endoscopy [146].

Traditional white-light endoscopy has limited sensitivity (50–78.4%) in detecting villous atrophy in CD, prompting the exploration of advanced endoscopic techniques. Among these, the water immersion technique shows high sensitivity (85–90.9%) and specificity (99–99.5%), while dye-based chromoendoscopy offers an impressive sensitivity of 94% and specificity of 99%. Other advanced methods, such as magnification endoscopy (sensitivity 86.4–95%, specificity 74.4–99%) and narrow band imaging (sensitivity 93%, specificity 95%), also demonstrate enhanced diagnostic accuracy. Capsule endoscopy (CE), with a sensitivity of 89% and specificity of 95%, is another valuable tool for diagnosing celiac disease, particularly when standard endoscopy fails to yield definitive results. CE plays a crucial role in the diagnosis of celiac disease, particularly in patients with positive serology and gastrointestinal symptoms but inconclusive or misdiagnosed histology, as well as in those with refractory celiac disease and for the diagnosis of complications of CD. These novel techniques significantly improve the detection of villous atrophy, offering promising alternatives to traditional methods [146].

### 6.5. Histology

CD primarily affects the small intestinal mucosa, with IELs typically exceeding 25 per 100 enterocytes, correlating with the severity of mucosal atrophy, though some cases may show normal IEL counts. Duodenal IELs are predominantly CD3^+^ and CD8^+^ T cells, detectable by immunohistochemistry. The lamina propria often exhibits increased cellularity, including plasma cells, lymphocytes, eosinophils, histiocytes, and mast cells, while neutrophils, crypt abscesses, and subtle crypt apoptosis may occasionally occur. Other characteristic changes include crypt hyperplasia and villous atrophy, the latter representing advanced but not pathognomonic mucosal damage. Histologic grading systems, such as the Marsh and modified Marsh–Oberhuber criteria and the simplified Corazza classification (grade A: isolated IEL increase; grade B: B1 partial villi, B2 complete villous loss), improve diagnostic consistency and inter-observer agreement. Accurate assessment of villous blunting requires examining 3–4 well-oriented villi in sequence to properly evaluate their architecture [147].

**Figure 7:**
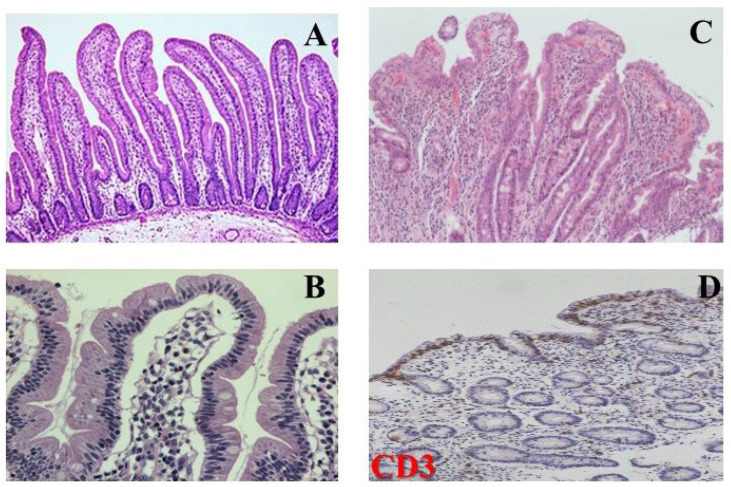
(**A**,**B**) normal intestinal mucosa. (**C**,**D**): Celiac Disease: Disturbance of villus/crypt ratio; Grading of villus widening/atrophy 3a, 3β, 3c, Crypt hyperplasia, Intraepithelial lymphocytosis > 25/100 enterocytes; Lamina propria infiltration with increased plasma cells, Lymphocytes CD3+, CD8+, 90% TCR αβ+ 10% γδ+. Images of mucosa courtesy of Kalliopi Stefanaki, M.D., PhD.

The histological findings seen in celiac disease appear in Figure 7.

### 6.6. Seronegative Celiac Disease

Seronegative CD poses a diagnostic challenge, as patients exhibit villous atrophy and gluten-responsive enteropathy despite negative conventional serologic markers such as anti-tissue transglutaminase and anti-endomysial antibodies. Diagnosis requires compatible histology, *HLA-DQ2* or *HLA-DQ8* or *DQ7.5* positivity, exclusion of other causes of villous atrophy, and clinical and histological response to a GFD. Consensus guidelines, including the Paris criteria, provide standardized definitions to differentiate seronegative CD from other non-celiac enteropathies, reducing the risk of misdiagnosis and unnecessary lifelong dietary restrictions. Although less common than seropositive CD, seronegative disease can carry similar morbidity [148]. In a 20-year multicenter cohort, patients with true seronegative CD had more severe symptoms at diagnosis and higher risks of complications and mortality, comparable to those with IgA deficiency. Age at diagnosis, classical presentation, and lack of response to a GFD were predictors of adverse outcomes, highlighting the importance of thorough evaluation and close clinical and histologic follow-up [149].

## 7. Who Should Be Tested for CD

Clinical indications for testing CD in adults and children, based on current guidelines from prominent gastroenterology and pediatric organizations, are integrated in Table 3.

## 8. Treatment

### 8.1. Gluten-Free Diet (GFD)

Based on the latest guidelines (2025 ESsCD for adults [135], and ESPGHAN 2020 for children [135], the primary treatment for CD remains a lifelong GFD, which is the only effective way to manage the condition. By completely eliminating gluten from the diet, individuals with CD allow the intestinal mucosa to heal and prevent further intestinal damage. Patients must avoid all foods containing wheat, barley, rye, and any products made with these grains, such as bread, pasta, and cereals. In addition, great care must be taken to prevent cross-contamination, as even trace amounts of gluten can trigger symptoms and cause harm. The term “gluten-free” applies to foods that meet strict limits on gluten content, as regulated by authorities based on safety data for individuals with celiac disease and wheat allergies. A recent review concluded that a daily intake of 50 mg of gluten is generally safe, although some individuals may experience mucosal changes with as little as 10 mg per day. However, the available studies are limited and heterogeneous, including both pediatric and adult populations [150].

Support from a multidisciplinary team, including a physician (pediatric gastroenterologist, gastroenterologist, or pediatrician) and an experienced dietitian, is essential for managing celiac disease. Regular follow-up is crucial to monitor dietary adherence, nutritional status, and to provide personalized guidance and support. Additionally, the use of new technologies, group education sessions, and involvement in regional patient societies can enhance the learning process and improve adherence to the gluten-free diet [150].

### 8.2. Adherence to GFD

Adherence to GFD in CD is challenging to assess due to varying definitions and subjective methods. A combination of tissue transglutaminase-IgA tests and questionnaires is recommended. However, negative anti-tTG results do not always indicate good adherence to the GFD and poorly correlate with recovery of intestinal damage [151]. Clinical improvement suggests adherence but does not confirm mucosal healing. Dietary interviews are informative but time-consuming, while self-reported questionnaires are less sensitive in children but useful for adolescents [150].

Gluten immunogenic peptides (GIPs) are undigested gluten fragments found in stool and urine, serving as noninvasive biomarkers to monitor adherence to a GFD in CD patients. GIPs can be detected in stool for up to 4 days and in urine within 4–6 h of gluten ingestion, with levels reflecting the amount of gluten consumed. GIPs are useful even in asymptomatic patients, as they can indicate ongoing intestinal damage. Various assays, including lateral flow tests for at-home use and ELISA tests for lab analysis, are available for detecting GIPs, helping patients track potential gluten exposure and improve GFD compliance [151].

A recent study assessed stool gluten GIPs to monitor gluten intake in celiac disease patients [152]. After a low-dose gluten challenge, stool GIP was highly sensitive to gluten exposure, with 100% sensitivity for doses of 250 mg or more. GIP levels peaked 12–36 h after ingestion and remained detectable for up to 5 days. Urine GIP showed poor sensitivity compared to stool. The findings suggest stool GIP testing is more reliable than urine or traditional methods for detecting low-level gluten exposure, though further research on patient behavior and assay specificity is needed [150,151].

### 8.3. Non-Dietary Therapies for Celiac Disease

CD treatment is evolving beyond a strict GFD, which, while effective, can be socially and psychologically challenging. Emerging non-dietary therapies—such as gluten sequestrants, transglutaminase inhibitors, and lymphocyte trafficking inhibitors—show promise in reducing gluten-induced symptoms and intestinal damage. Phase 2 studies are encouraging, but further Phase 3 trials are needed to confirm safety, efficacy, and long-term outcomes. Emerging therapies for celiac disease aim to complement or eventually reduce reliance on a strict gluten-free diet by targeting various points in disease pathogenesis, including enzymatic gluten degradation (e.g., orally administered proteases like latiglutenase and other endopeptidases to break down immunogenic gluten peptides) and barrier-enhancing agents such as larazotide acetate to reduce intestinal permeability and thus limit gluten peptide entry into the lamina propria. Other strategies focus on immune modulation and tolerance, including therapies designed to retrain the immune system to tolerate gluten antigens (e.g., antigen-based immune tolerizing agents like KAN-101) or inhibit pro-inflammatory pathways (e.g., IL-15 inhibitors like TEV-53408 in clinical development) and transglutaminase inhibitors to prevent gluten modification that enhances immunogenicity. Additional approaches under investigation include gluten sequestrants, microbiome modulation, and nanoparticle-based immune therapies, with many agents in phase I–III trials but none yet approved to replace dietary management. These innovations collectively represent a shift toward more flexible, targeted management of celiac disease beyond diet alone [153]. Personalized approaches, considering genetic, immunological, and microbiological factors, may optimize treatment. Multidisciplinary care, ethical considerations, and economic feasibility are also crucial as these therapies move toward clinical use. Future research will focus on long-term safety, adjunctive treatments, and novel therapeutic targets to improve the quality of life for CD patients [154].

## 9. Complications

### 9.1. Refractory Celiac Disease (Persistent Symptoms Despite Strict Diet)

Fewer than 1% of CD patients develop refractory CD (RCD), making it a rare condition that most gastroenterologists may not frequently encounter [56]. However, understanding the basics of RCD and non-responsive CD (NRCD) is essential, as RCD can lead to severe complications, including high mortality from intestinal failure, enteropathy-associated T-cell lymphoma (EATL), and life-threatening infections. Diagnosing RCD requires complex workups, often involving specialized labs and referral centers, particularly in differentiating between RCD type I (RCDI) and type II (RCDII) [3,17,56,155].

RCD is characterized by persistent symptoms such as malabsorption and villous atrophy despite strict adherence to a gluten-free diet for at least one year. It is more common in adults and is categorized into two types: RCDI, which is less severe, and RCDII, which carries a higher risk of progressing to EATL. Diagnosis involves ruling out other causes, such as gluten contamination, and confirming the presence of genetic markers (*HLA-DQ2*/*DQ8*) along with positive celiac serology. Although the prevalence of RCD has decreased, it remains a complex condition to diagnose and manage, with RCDI responding better to immunosuppressive treatments than RCDII, for which treatment options are more limited and offer only temporary relief [3,17,56,155,156]. Treatment of refractory celiac disease (RCD) is challenging, with no definitive cure. For RCD type I, first-line treatment includes glucocorticoids like open-capsule budesonide, and immunomodulators such as azathioprine may be used if needed. RCD type II is harder to treat, with options like cladribine or autologous stem cell transplantation for severe cases. New therapies, such as biologics, show limited effectiveness [3,17,56,155,156].

### 9.2. Celiac Disease and Cancer

In CD, the risk of malignancy is generally low but higher for gastrointestinal cancers, especially small bowel adenocarcinoma (SBA) and enteropathy-associated T-cell lymphoma (EATL). A study found that 3.7% of CD patients developed malignancies, with SBA being the most common. Risk factors include delayed diagnosis, untreated CD, and persistent villous atrophy. Early diagnosis and adherence to a gluten-free diet (GFD) are crucial for preventing these complications. B-cell lymphoma, particularly diffuse large B-cell lymphoma (DLBCL), is also a concern. Although malignancies are rare, they often have a poor prognosis, highlighting the importance of close monitoring and early intervention [157].

CD is a chronic inflammatory disorder triggered by gluten in genetically predisposed individuals, causing duodenal inflammation. Beyond the gut, CD is linked to an increased risk of cancers, including intestinal cancers, lymphomas, and oropharyngeal cancers. In a cohort of 1757 Italian celiac patients followed for an average of 18 years, six developed papillary thyroid carcinoma, corresponding to a 2.5-fold increased risk. Age at celiac diagnosis and gluten intake did not influence cancer occurrence, suggesting that celiac disease may increase the risk of thyroid malignancy independently of diet or early diagnosis [158]. However, in a large Swedish population-based study of 29,074 individuals with biopsy-confirmed celiac disease, there was no evidence of increased risk for thyroid cancer, including papillary thyroid cancer, compared with matched controls [159]. This risk of cancer development may be related to common cancer hallmarks present in CD patients. Research into gut microbiota, microRNAs, and DNA methylation aims to uncover connections between CD and cancer, but the evidence remains inconsistent. This review highlights the genomics and molecular factors linking CD to cancer [160].

## 10. Prevention Strategies

Environmental factors, such as viral infections, may trigger CD by increasing gut permeability or causing dysbiosis and inflammation, leading to autoimmune-like responses. Early studies suggested introducing gluten between 4 and 6 months during breastfeeding, but later trials found no protective effect from delayed or limited gluten introduction in the first year. Ongoing trials, including PreCiSe and GRAIn, are evaluating whether prolonged gluten restriction or targeted probiotic supplementation during the first 3–5 years can reduce CD risk in genetically susceptible children. These findings suggest that combining infant diet and microbiome modulation may offer potential for primary prevention, although long-term evidence is still needed [34].

## 11. Conclusions

In conclusion, CD is a complex, multifaceted disorder that extends beyond the gastrointestinal system, presenting with a wide range of symptoms and extraintestinal manifestations. Advances in understanding its genetic, immune, and environmental factors have provided valuable insight into its pathogenesis. Affecting approximately 1.4% of people worldwide, both adults and children, the diagnosis is usually straightforward, relying on serologic testing, duodenal biopsy, genetic analysis, and clinical response to a gluten-free diet. However, some patients do not respond adequately, requiring further evaluation to rule out serious complications such as refractory CD or other intestinal pathology.

Increasing awareness of the disease’s prevalence and diverse presentations, along with the availability of accurate serologic and emerging diagnostic tests, can enhance early screening, assess adherence to a GFD, and identify non-responders to prevent complications, including nutritional deficiencies, osteoporosis, refractory celiac disease, and malignancies. While the gluten-free diet remains the cornerstone of management, pharmacologic therapies are under development. A deep understanding of this complex disease is essential for optimizing diagnosis, management, and long-term patient care, underscoring the ongoing need for research.

## Figures and Tables

**Table 1:** High-risk groups for CD development.

High-Risk Group
First-degree relatives
Second-degree relatives
Type 1 diabetes (children)
Autoimmune thyroiditis
Juvenile idiopathic arthritis
Autoimmune hepatitis
Selective IgA deficiency
Down syndrome
Turner syndrome
Williams syndrome

**Table 2:** The classical symptoms of celiac disease in children and adults.

Symptom Type	Children (Classic)	Adults (Classic)
**Gastrointestinal**	Chronic diarrhea, abdominal bloating, distention, steatorrhea, vomiting	Chronic diarrhea or constipation, bloating, abdominal discomfort, steatorrhea (less prominent than in children)
**Growth/Nutrition**	Failure to thrive, weight loss, short stature, delayed puberty	Weight loss (may be mild), anemia, nutrient deficiencies (iron, folate, B12, vitamin D)
**Systemic**	Fatigue, irritability, pallor	Fatigue, weakness, iron-deficiency anemia, osteoporosis/osteopenia

**Table 3:** Who should be tested for Celiac Disease?

When/Why to Test	Adults (ESsCD 2025) [137]	Children/Adolescents (ESPGHAN 2020) [135]
**Symptoms suggestive of CD (gastrointestinal or malabsorption)**	GI symptoms (chronic diarrhea/steatorrhea, weight loss, bloating, abdominal pain), malabsorption, iron-deficiency, steatorrhea, etc.	Chronic or intermittent diarrhea/constipation, recurrent vomiting/nausea, chronic abdominal pain or distension, unexplained growth failure or failure to thrive.
**Extra-intestinal signs/non-classical manifestations** (e.g., anemia, bone disease, liver, skin, neuro)	Unexplained iron-deficiency anemia, osteopenia/osteoporosis, unexplained elevated liver enzymes, dermatitis herpetiformis, neurological symptoms, etc.	Iron-deficiency anemia, short stature, delayed puberty, bone demineralization/fractures, dental enamel defects, recurrent aphthous stomatitis, persistent fatigue/irritability, skin manifestations like dermatitis, unexplained liver abnormalities, etc.
**High-risk/at-risk groups** **(even if asymptomatic** **or mild symptoms)**	First-degree relatives of people with CD; people with associated conditions (e.g., certain autoimmune diseases) or other risk factors, especially if clinical context suggests it. The 2025 ESsCD also emphasizes a “case-finding” rather than mass population screening.	First-degree relatives with CD; children/adolescents with conditions associated with increased CD risk, e.g., type 1 diabetes, autoimmune thyroid disease or autoimmune liver disease, certain genetic syndromes (trisomies/chromosomal disorders as per national guidance), IgA deficiency or other immunologic risks.

## Data Availability

The original contributions presented in this study are included in the article. Further inquiries can be directed to the corresponding author.

## Abbreviations

The following abbreviations are used in this manuscript:

HLA	Anti-human leukocyte antigen
AC	Ahead of Christ
CD	Celiac disease
GWAS	Genome-wide association studies
MHC	Major histocompatibility complex
SCFA	Short chain Fatty acids
AoR	Adjusted Odds Ratio
INF	Interferon
ESPGHAN	European Society of Pediatric Gastroenterology, Hepatology and Nutrition
IELS	Intraepithelial lymphocytes
IL	Interleukin
NKG2D	Natural Killer Group 2, Member D
IgA	Immunoglobulin A
tTG	tissue transglutaminase
ADHD	Attention-Deficit/Hyperactivity Disorder
ASD	Autism spectrum disorders
DED	Dental enamel defects
GFD	Gluten-free diet
RAS	Recurrent aphthous stomatitis
MRI	Magnetic Resonance Imaging
DH	Dermatitis Herpetiformis
DEXA	Dual-Energy X-ray Absorptiometry
FRAX	Fracture Risk Assessment Tool
CCM	Celiac Cardiomyopathy
PH	Pulmonary Hemosiderosis
IgAN	IgA Nephropathy
FVAS	Facial Visual Analogue Scale
FSS	Fatigue Severity Scale
NAFLD	Non-Alcoholic Fatty Liver Disease
AIH	Autoimmune Hepatitis
IBS	Irritable Bowel Syndrome
aHR	Adjusted Hazard Ratio
EoE	Eosinophilic Esophagitis
EMA	Anti-Endomysial Antibodies
AGA	antigliandin antibodies
DGP	Deamidated Gliadin Peptides
POCT	Point-of-Care Test
ESsCD	European Society for the Study of Coeliac Disease
GIPs	Gluten Immunogenic Peptides
RCD	Refractory Celiac Disease
